# The Role of Optimism and Self-Efficacy in the Relationship between Academic Stress and Depressive Symptoms in Medical Students Including the Use and Knowledge of Structural Health Promotion Offers

**DOI:** 10.1007/s40670-024-02240-4

**Published:** 2024-12-18

**Authors:** Annika Arnold, Petra Maria Gaum, Jessica Lang

**Affiliations:** Institute for Occupational, Social and Environmental Medicine, Medical Faculty, RWTH Aachen University, Aachen, Germany

**Keywords:** University health promotion offers, Medical students, Academic stress, Depressive symptoms, Optimism, Self-efficacy

## Abstract

**Background:**

In recent years, studies have highlighted that stress levels among medical students are alarmingly high. The study aim was to examine how academic stress and depressive symptoms in medical students are affected by individual moderators (self-efficacy/optimism) and structural influencing factors (health promotion offers).

**Methods:**

Data collection occurred at three measurement points during the first 1.5 years at a medical school in Germany [baseline measurement (BM; Winter 2019, 226 participants), follow-up measurement 1 (FUM1; Summer 2020, 106 participants) and follow-up measurement 2 (FUM2; Winter 2020, 107 participants)]. Cross-sectional and longitudinal analyses were conducted with the data of FUM1 and FUM2, BM being only cross-sectionally analyzed as a baseline measurement. Interactions were calculated cross-sectionally with multiple linear regressions, and longitudinally with mixed effects models.

**Results:**

Academic stress was positively correlated with depressive symptoms cross-sectionally. In the longitudinal sample, optimism was found to moderate the association between academic stress and depressive symptoms, while the moderating effect of self-efficacy was found cross-sectionally for FUM2. “University sports offers” was the most-used health promotion offer for both FUM1 and FUM2. The other services, especially “psychological counseling”, were rarely used by the students. The prevalence of depressive symptoms increased during the study course (BM: 4.9%; FUM1: 25.5%; FUM2: 23.4%).

**Conclusion:**

The increase of depressive symptoms linked to rising academic stress within the study course is alarming. Individual attributes such as optimism and self-efficacy have a moderating role on the relationship between academic stress and depressive symptoms and prevention offers may affect those individual attributes.

.

**Supplementary Information:**

The online version contains supplementary material available at 10.1007/s40670-024-02240-4.

## Background

Depression is defined as a mental health condition associated with core symptoms such as depressed mood, reduction in drive, loss of interest, and additional signs such as a reduction in the ability to concentrate, reduced self-confidence, feelings of guilt or sleep disturbances [1]. Compared to the total German population, medical students suffer more frequently from mental illnesses such as depression and stress-related disorders [2]. This is in line with a finding from Hope et al. (2014) [3], which indicates that the prevalence rate of depression and anxiety is higher among medical students than among young adults of the same age. In addition, the prevalence rate of suicide ideation is high among medical students (11.2%) compared to the same age group in the general population (6.9% [4]). An increased prevalence of depression among medical students was also found internationally. In Southern Ethiopia, close to one-third of all medical students (30%, which was higher than among the general population) were found to suffer from mental distress [5]. A similarly high prevalence rate of depression was found among medical students in Cameroon (30.6% [6]). More recently, researchers in Northern Peru have found seven out of ten medical students to show depressive and anxious symptoms [7]. A similar prevalence rate (72%) of depression among medical students has been found in China [8], with the global rate of depression in medical students being 28% according to Puthran et al. (2016) [9].

Medical training involves a lot of psychological stress for medical students. There is much research outlining the alarmingly high levels of perceived stress and elevated rates of depression among medical students (e.g., [3, 10–14]). The role of academic stressors (such as taking part in examinations, the students’ performance, and lack of time for revisions) is supported by several studies, highlighting them as the main sources of stress in medical students [15–17]. The experience of stress has been shown to have a negative influence on individuals’ psychological and physical health [18], making them susceptible, for instance, to a greater risk of cardiovascular diseases [19]. For medical students, stress is not only a health hazard in itself, but it can also lead to a greater health impairment in the form of a depressive disorder [20]. A link between their perceived stress and the development of depressive symptoms can therefore be conjectured.

The assumption that there is an association between stress and mental health has already been examined. Hamesch et al. (2014) [21] have found rumination to be a link between stressors in dental students and their psychological health (depression in particular). Karyotaki et al. (2020) [22] have observed a strong association between common psychological disorders and perceived stress in different areas of life among first-year college students in nine countries. The stress factors associated with major depressive disorders have been found to be those concerning financial situations, relationships at work/school and relationships within the family. Based on the relationship between stress and depression, our study examines the individual factors optimism and self-efficacy as potential influencing factors both cross-sectionally and longitudinally.

As early as 1992, Scheier and Carver [23] underlined the effect of optimism on people’s well-being. They defined a personality trait called dispositional optimism as “the generalized, relatively stable tendency to expect good outcomes across important life domains” [24]. Their work revealed that optimists, in contrast to pessimists, experience less subjective stress in stressful situations, differing in their ability to cope with stress [23]. Recent studies also report that optimism has a great effect on people’s well-being [25, 26]. Shi et al. (2016) [26] have found factors such as optimism, resilience and hope to correlate negatively with depressive symptoms in Chinese medical students. Optimism has been identified as a moderator on the relationship between stress and depression in several studies involving samples of workers [27, 28] and college students [29, 30].

Alongside optimism, self-efficacy as a personal resource seems to play an important role in the context of the development of stress and depressive symptoms. Defined as “people’s beliefs about their capabilities to produce effects” [31], it influences their feelings, thoughts and behavior given the relevance of this “belief” to how individuals perceive themselves. Several studies have concluded that self-efficacy has a stress buffering effect on depressive symptoms (e.g. [32]), and is, therefore, an important resource for life satisfaction [33, 34]. Optimism and self-efficacy have been described as “significant predictors of coping, health and emotions” by Schwarzer in 1994 [35], with both traits having been found to partially function as mediators in the association between life satisfaction and depressive symptoms in Chinese medical students [8].

Hence, it stands to reason that both personal characteristics play a crucial role in the context of stress, depression and possible interventions. However, to our knowledge, the moderating role of neither optimism nor self-efficacy in the association between academic stressors and depressive symptoms has yet been examined in medical students. Thus, the present study investigates a potential stress buffering effect of both individual attributes cross-sectionally and longitudinally.

In addition to the individual factors, we investigated various health promotion offers as structural factors. Many studies have examined coping strategies as behavioral prevention measures (e.g., [2, 36–39]), while research about external health promotion offers is still at a nascent stage. Focusing on these external offers is important so that the responsibility for the preventive tasks does not lie exclusively with the individual.

Many companies have established prevention services. As most people spend a large proportion of their time at work, the workplace is an important setting for health promotion [40, 41]. The goal of organizational health promotion is to reduce health risks and increase the quality of life for workers [41, 42]. The protection of employees’ health becoming increasingly important for employers, there is a tendency, according to Tetrick et al. (2015) [43], toward implementing stress management interventions as part of a workplace wellness program. In our study, by focusing on medical students, we have sought to transfer the idea of external health promotion offers to the university context.

Bitonte and DeSanto (2014) [44] underline the importance of integrating time for physical activity in the medical curriculum based on the premise that the improvement of general health among medical students can help prevent mental illnesses. Another way to approach the promotion of mental health is psychological counseling. However, the fear of embarrassment (appearing weak to others) and the lack of awareness about the available support services can keep students from seeking help in the university environment [45]. Givens and Tjia (2002) [46] report that the fear of stigma likely results in an undertreatment of depression in medical students. Psychological counseling, however, has been shown to be an important tool to promote mental well-being.

Several intervention studies have tested health promoting programs in universities [47–52]. Testing a mindfulness-based stress reduction (MBSR) program for first- and second-year medical students, and measuring their levels of satisfaction, Aherne et al. (2016) [47] found the program to be associated with high satisfaction levels when offered voluntarily. According to the authors, the most important aspects of a successful program involve paying attention to individual characteristics and creating a comfortable training environment in which participants do not feel judged. As regards changes in the curriculum and their potential impact [48, 53, 54], Slavin et al. (2014) [53] adjusted the medical training through the addition of courses for resilience and stress reduction, social events, changes in the grading system or the structure of particularly stressful courses, and found these alterations to lead to a reduction in study-related stress. This study therefore analyzes the knowledge and use of various health promotion offers and identifies possible correlations with the mental well-being of students to underscore the importance of structural offers to improve mental health.

The mental well-being of medical students is subject to change throughout their training. Ludwig et al. [55] have found that the number of medical students at risk of depression had risen after the third year of study (39%) compared to the first year (28.4%), with the perceived stress level having been augmented in these students (first year: 5.51; third year: 6.49). Possibly, this could be due to the risings demands placed on medical students in the course of their studies. In order to take the possible time-related variation into account, we will assess the change in prevalence of academic stress and depressive symptoms over the first three semesters of medical training.

### Study Aim

The primary objective of this study is to examine academic stress and its impact on depressive symptoms considering the moderating influence of individual factors in medical students. Optimism and self-efficacy are the individual influencing factors examined in this regard. In an exploratory approach a second goal of the study is to consider possible structural factors, such as the students’ knowledge and use of university health promotion offers.

In addition, since past research mentioned variation of stress and depressive symptoms in the course of the study, we want to explore the results in the context of time.

According to past research on academic stress and depressive symptoms and the psychological constructs of optimism and self-efficacy, we are able to deduct three hypotheses considering the direct and moderating relationships therebetween. Since there is a lack of research on the impact of structural interventions and the variations of stress and depressive symptoms over time in the area of our interest, we are not able to deduce respective hypotheses for these two additional factors. Instead, we formulate two exploratory research questions to bring research forward.

Hypothesis 1 postulates a positive association between academic stress and depressive symptoms. That means that the more students report experiencing academic stress, the more they are likely to also report depressive symptoms. In the moderator hypotheses, the effects of optimism (hypothesis 2) and self-efficacy (hypothesis 3) on the relationship between academic stress and depressive symptoms are tested. In hypothesis 2, it is assumed that the relationship between academic stress and depressive symptoms is less pronounced in students reporting higher levels of optimism. For self-efficacy (hypothesis 3), higher levels of self-efficacy are expected to weaken the relationship between academic stress and depressive symptoms. The study analyzes all three hypotheses cross-sectionally (hypotheses 1A, 2A, 3A) and longitudinally (hypotheses 1B, 2B, 3B). These two approaches to analyzing our data complement and strengthen the observed relationships, as our cross-sectional analyses show results for specific time points and trends over time are investigated with our longitudinal analyses.

Research question 1 is aimed at descriptively examining the students’ knowledge about and use of health promotion activities offered by the university as structural influencing factor on depressive symptoms.

Research question 2 descriptively presents the prevalence of depressive symptoms and academic stress during the first three semesters of medical training.

## Methods

### Design and Setting

The data for this study, including both the cross-sectional and longitudinal parts, were collected at three different measurement points in the first one and a half years of a German medical school program. The study had a within-subjects design with one-semester time lag between the different cross sections. The first survey (baseline measurement; BM), a non-mandatory paper–pencil test, was conducted after a mandatory paper–pencil test in October 2019. For the second and third surveys, an online questionnaire was used due to the pandemic-related restrictions. The second survey will be henceforth referred to as “follow-up measurement 1” (FUM1) and the third one as “follow-up measurement 2” (FUM2). FUM1 was provided via SoSciSurvey from 25th of May to 28th of July 2020 and FUM2 in November 2020. The link was distributed via e-mail through the students’ first-year coordinator, an official social media platform used by the local university students and a university internal online event.

The BM took place before the start of the pandemic, with FUM1 and FUM2 being organized subsequently. Given that at BM the sample was different from both FUM1 and FUM2, the research question 1 and hypotheses are calculated only with respect to FUM1 and FUM2, hypothesis 1A and research question 2 being an exception where we also used the data of BM. A study design such as this enables a cross-sectional analysis that focuses on the relationship between different variables at a specific time point, while the longitudinal analysis shows trends over time, leading to a more causal interpretation of the results. This ensures that the results are not dependent on the time of data collection. The aim of using these two approaches to analyze our data was to gain a comprehensive insight and to foster the generalizability of the findings. At the beginning of each questionnaire at each measurement point, all participants created an individual code, which was used to recognize their answers anonymously at each measurement point. The code cannot be traced back to an individual as it was generated out of various letters and numbers (for example, the first letter of the mother’s/father’s first name, the mother’s birth month, etc.). Participants provided informed consent prior to their participation. The study was approved by the local ethics commission of the study center.

### Variables

**Fig. 1:**
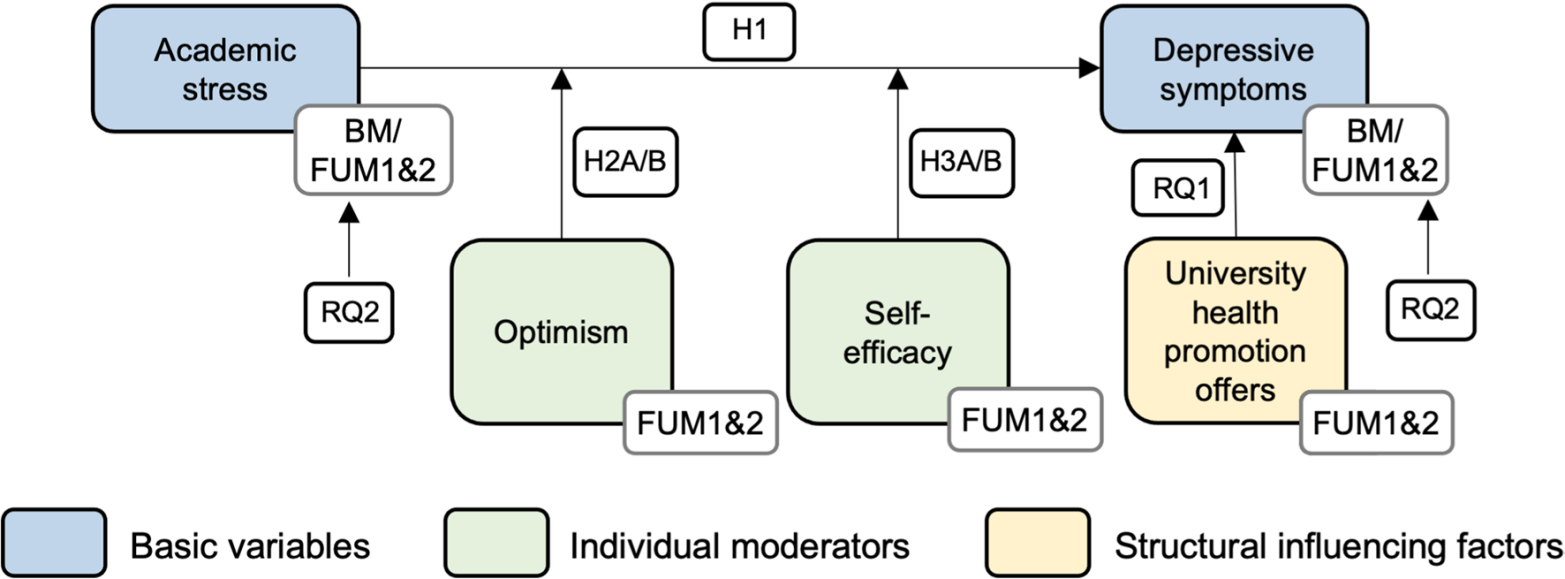
Visualization of the collected variables at baseline measurement (BM) and both follow-up measurements (FUM1&2). H1 = hypothesis 1, H2A/B = hypothesis 2A/B, H3A/B = hypothesis 3A/B, RQ1 = research question 1, RQ2 = research question 2

An overview of the variables examined at baseline, as well as the two follow-up measurement points is given in Fig. 1. The figure also shows an overview of the postulated hypotheses and the two research questions.

#### Academic Stress

Academic Stress was measured by the Medical Student Stressor Questionnaire (MSSQ [17]), which measures six different domains of stress in medical studies. In this study, we focused only on the domain of academic stress. The items were translated from English into German and adapted to medical studies in Germany.

A short scale was created with five items of the MSSQ to compare the variable “academic stress” at BM with FUM1 and FUM2. The descriptive results are shown in Table 1. Cronbach’s alpha for BM was 0.81. In FUM1 and FUM2, in addition to the short scale, we used 11 of the 13 items for a long scale of academic stressors. On a Likert scale with five points (from 0 = “no stress at all” to 4 = “severe stress”), students were required to indicate how much stress they experience because of aspects such as “high amount of learning” or “competitive learning environment”. For academic stress, Cronbach’s alpha was 0.83 in FUM1 and 0.84 in FUM2. Mean values were used to create the scale.

**Table 1:** Description of study population at three different measurement points

	BM (Winter 2019)		FUM1 (Summer 2020)		FUM2 (Winter 2020)	
	M(SD)	PR (%)	M(SD)	PR (%)	M(SD)	PR (%)
**Age**	20.94 y (3.68 y)		20.90 y (3.38 y)		21.51 y (3.2 y)	
**Depressive Syndrome (PHQ),** sum scale	3.47 (3.33)	4.9	7.6 (4.95)	25.5	6.83 (5.1)	23.4
**Academic Stress** (short variable)	2.22 (0.82)		2.81 (0.77)		2.43 (0.89)	

*n* number of participants, *M* Mean, *SD* Standard Deviation, *PR* Prevalence Rate, *BM* baseline measurement, *FUM1* follow-up measurement 1, *FUM2* follow-up measurement 2, *BM n* = 226, *FUM1 n* = 106, *FUM2 n* = 107

#### Depressive Symptoms

The German Version of the Patient Health Questionnaire (PHQ-9 [56]) is a screening tool for the diagnosis of depression, which we used in this study to detect depressive symptoms. The nine items are based on the nine DSM-IV criteria for major depression. A four-point Likert scale was used to indicate symptoms (such as “little interest or pleasure in activities” or “tiredness or feeling of having no energy”) of the last two weeks (0 = “not at all” to 3 = “nearly every day”). On a sum scale, Cronbach’s alpha was 0.79 for BM, 0.84 for FUM1 and 0.85 for FUM2. To investigate the prevalence of a depressive syndrome within the sample, we created a dichotomous variable as described in the official manual [56]. For the dichotomous variable, students with a depressive syndrome were coded with “1” and those without a depressive syndrome with “0”. The criteria for being elected for group “1” (any depressive syndrome) were, among other aspects, having answered at least two specific questions (including item 1 and/or item 2) with "more than half of the days".

#### Optimism and Self-efficacy

The short version of the Self-efficacy-optimism–pessimism questionnaire (SWOP-K9) was used to assess the students’ self-efficacy and optimism [57]. Optimism was measured with two items (e.g., “I always see the good side of things”) and self-efficacy with five items (e.g., “If someone resists me, I find ways and means to assert myself.”). The students were asked to indicate on a four-point Likert scale (from 1 = “does not apply” to 4 = “does apply”) whether the statements applied to their attitudes and feelings. Since the scale for optimism consists of two items, the correlations were calculated to assess the internal consistency. For FUM1 and FUM2, it was r_FUM1=_ 0.70 and r_FUM2_ = 0.70, respectively. Cronbach’s alpha for self-efficacy was 0.67 for FUM1 and 0.64 for FUM2.

#### University Health Promotion Offers

The participants were asked about their knowledge and use of various health promoting offers at their university. The possible answers were “Yes, I know about it”, “Yes, I use it” and “No, I do not use or know it”. The various offers were divided into three categories: University sports offers, psychological counseling and study-related stress management offers (see also Table 5). University sports offers include a wide range of over 100 different sports courses of the university (team sports as well as fitness courses such as yoga, boxaerobic, spinning or exercise center).

##### Statistical Analysis

As a first step, we performed a correlation analysis with the main demographic study variables (age, gender), to identify possible confounding variables. As no relevant correlations of any significance appeared in this analysis (see supplementary Table 1, Additional File 1), these variables were not controlled for in the subsequent analyses. For the key variables, a correlation analysis was performed using Spearman’s rank correlation coefficients due to data that were not normally distributed.

To examine the cross-sectional hypothesis 1A, a linear regression with stress as the predictor and depressive symptoms as the outcome variable was performed.

The interaction hypotheses 2 and 3 for optimism (hypothesis 2) and self-efficacy (hypothesis 3) were calculated with the SPSS Macro PROCESS for Apple, which is based on multiple linear regression. Stress was included as the predictor, depressive symptoms as the outcome and optimism and self-efficacy were entered as moderators, respectively [58].

In the longitudinal analysis, a mixed effects model was calculated to control for the random effect of time on the tested association in hypotheses 1B, 2B and 3B. For this purpose, R (version 4.0.3 (2020–10-10)) and R Studio (version 1.4.1103 [59]) with the package “lme4” and “lmerTest” for Apple were used [60–62].

To investigate research question 1, a descriptive analysis was carried out to determine, in percentage terms, the frequency of the students’ reporting of knowledge or use of health promotion offers at their university. Further, correlation analyses in each FUM were performed to investigate relations between the different types of health promotion offers and depressive symptoms.

For research question 2, the prevalence of depressive symptoms and academic stress was described over the course of the first three semesters.

For all other analyses, SPSS 25 for Apple was used [63]. As level of significance, a p-value lower than 0.05 was defined.

## Results

### Study Population

The BM involved 242 participants in total. After the exclusion of students due to unintended semesters or incomplete questionnaires, 226 students were left, of whom 77 (34.1%) were male and 149 (65.9%) female. A total of 297 students started their medical studies in this semester, with a response rate of 76.1%.

At the second measurement point (FUM1), a total of 139 participants started the online questionnaire and 108 of them finished it. Two questionnaires were excluded because the students were not in their first year. Thus, the final sample consisted of 106 medical students (response rate of 38.4%) with 27 male (25.5%) and 79 female students (74.5%).

The third online survey (FUM2) was finished by 111 participants. Again, after excluding students from unintended semesters, 107 surveys (response rate of 39.8%) were left, of which 33 were male (30.8%) and 74 female (69.2%).

The female-to-male ratio among students reflects the situation at medical schools in Germany [64].

### Descriptive Results

The descriptive results of the potential control, predictor and criterion variables are shown in Table 1.

The cross-sectional analyses 226 (BM; only hypothesis 1A) included 106 (FUM1) and 107 (FUM2) medical students, while 43 medical students, who could be matched according to their code on both measurement occasions, were included in the longitudinal analysis. BM with the larger sample before the pandemic was only considered for the analysis of hypothesis 1A and research question 2 because the composition of the sample was different. Table 2 shows a more detailed description of the key variables “depressive symptoms”, “academic stress”, “optimism” and “self-efficacy”. Further, the results of the correlation analysis using Spearman’s rank correlation coefficients are presented in Table 3.

**Table 2:** Description of study population for cross-sectional and longitudinal analyses

		FUM1			FUM2		
	Ref	M(SD)	Mdn	Range	M(SD)	Mdn	Range
**Depressive symptoms**							
Cross-sectional^1^	0–27	7.60 (4.95)	7.00	0–24	6.83 (5.10)	5.00	0–24
Longitudinal^2^	0–27	7.26 (4.81)	6.00	1–18	6.26 (4.73)	5.00	0–19
**Academic Stress**							
Cross-sectional^1^	0–4	2.06 (0.66)	2.09	0–4	1.90 (0.73)	2.00	0–4
Longitudinal^2^	0–4	1.86 (0.62)	1.91	0–4	1.73 (0.63)	1.73	0–4
**Optimism**							
Cross-sectional^1^	1–4	2.93 (0.79)	3.00	1–4	2.91 (0.77)	3.00	1–4
Longitudinal^2^	1–4	2.92 (0.77)	3.00	1–4	2.97 (0.78)	3.00	1–4
**Self-efficacy**							
Cross-sectional^1^	1–4	2.70 (0.48)	2.70	1–4	2.74 (0.46)	2.80	1–4
Longitudinal^2^	1–4	2.79 (0.47)	2.80	1–4	2.78 (0.44)	2.80	1–4

*n* number of participants, *Ref* Reference, *M* Mean, *SD* Standard Deviation, *Mdn* Median, *FUM1* follow-up measurement 1, *FUM2* follow-up measurement 2, ^1^ = n_FUM1_ = 106, n_FUM2_ = 107. ^2^ = n_FUM1+FUM2_ = 43

**Table 3:** Spearman’s rank correlation coefficients (FUM1: *n* = 106; FUM2: *n* = 107)

	FUM1				FUM2			
	1)	2)	3)	4)	1)	2)	3)	4)
**1) Depressive symptoms**	-				-			
**2) Academic Stress**	0.63*	-			0.50**	-		
**3) Optimism**	−0.47**	−0.38**	-		−0.40**	−0.37**	-	
**4) Self-efficacy**	−0.36**	−0.54**	0.48**	-	−0.37**	−0.51**	0.43**	-
**5) University sports offers**								
**Knowledge**	0.08	0.22*	−0.08	−0.126	0.02	−0.09	−0.00	−0.03
**Use**	−0.02	−0.15	0.08	0.054	−0.05		0.08	0.05
**6) Psychological Counseling**								
**Knowledge**	0.08	0.09	−0.10	−0.09	−0.11	−0.14	0.10	0.17
**Use**	0.15	0.06	−0.07	0.03	0.14	0.21*	−0.20	−0.11
**7) Study-related stress management offers**								
**Knowledge**	−0.05	0.00	0.11	0.00	−0.27**	−0.18	0.22**	0.12
**Use**	−0.02	−0.01	−0.06	0.04	0.03	0.06	0.02	−0.01

*p*-value (significance; two-tailed), +  = 0.05 < *p*-value < 0.1, * *p*-value < 0.05; ** *p*-value < 0.01, *FUM1* follow-up measurement 1, *FUM2* follow-up measurement 2

The first hypothesis tested the positive relationship between academic stress and depressive symptoms cross-sectionally (hypothesis 1A) and longitudinally (hypothesis 1B). The regression analysis for BM, FUM1 and FUM2 separately shows that academic stress is a significant predictor for depressive symptoms (β_BM_ = 0.24, t_BM_ = 3.75, p_BM_ < 0.01; β_FUM1_ = 0.60, t_FUM1_ = 7.65, p_FUM1_ < 0.01; β_FUM2_ = 0.51, t_FUM2_ = 6.01, p_FUM2_ < 0.01). The overall model was also significant [BM: F(1,224) = 14.05, *p* =  < 0.01; FUM1: F(1,104) = 58.44, *p* =  < 0.01; FUM2: F(1,105) = 36.09, *p* < 0.01]. Academic stress explains 5.5% variance of depressive symptoms at BM, 35.4% variance at FUM1 and 24.9% variance at FUM2. The results of the longitudinal analysis with the data of FUM1 and FUM2 also indicated the relationship between academic stress and depressive symptoms to be significant (see first line in Table 4). Hypothesis 1B was thereby confirmed.

**Table 4:** Random effects model of the moderating effect of optimism and self-efficacy on the association between academic stress and depressive symptoms

DV: Depressive symptoms	Optimism			Self-efficacy		
	β	t	*p*	β	t	*p*
**Fixed effect**						
Academic Stress	0.26	3.0	< 0.01	0.43	3.8	< 0.01
Individual attributes (moderator)	−0.48	−5.7	< 0.001	−0.10	−0.9	0.38
Academic stressors*individual attributes	**−0.26**	**−3.4**	** < 0.01**	−0.21	−1.8	0.07
R^2^	0.46			0.24		
**Random effect** (for measurement occasion)						
Variance	0.00			0.00		
Standard deviation	0.00			0.00		
R^2^	0.00			0.00		

*β* = standardized regression coefficient,* t* = *t*-value,* p* = *p*-value (significance), *R*^*2*^ = *R*-squared (explained variance), DV = dependent variable, n = 43 medical students who participated in FUM1 and FUM2. FUM1 = follow-up measurement 1; FUM2 = follow-up measurement 2. Significant interaction terms are written in bold

**Fig. 2:**
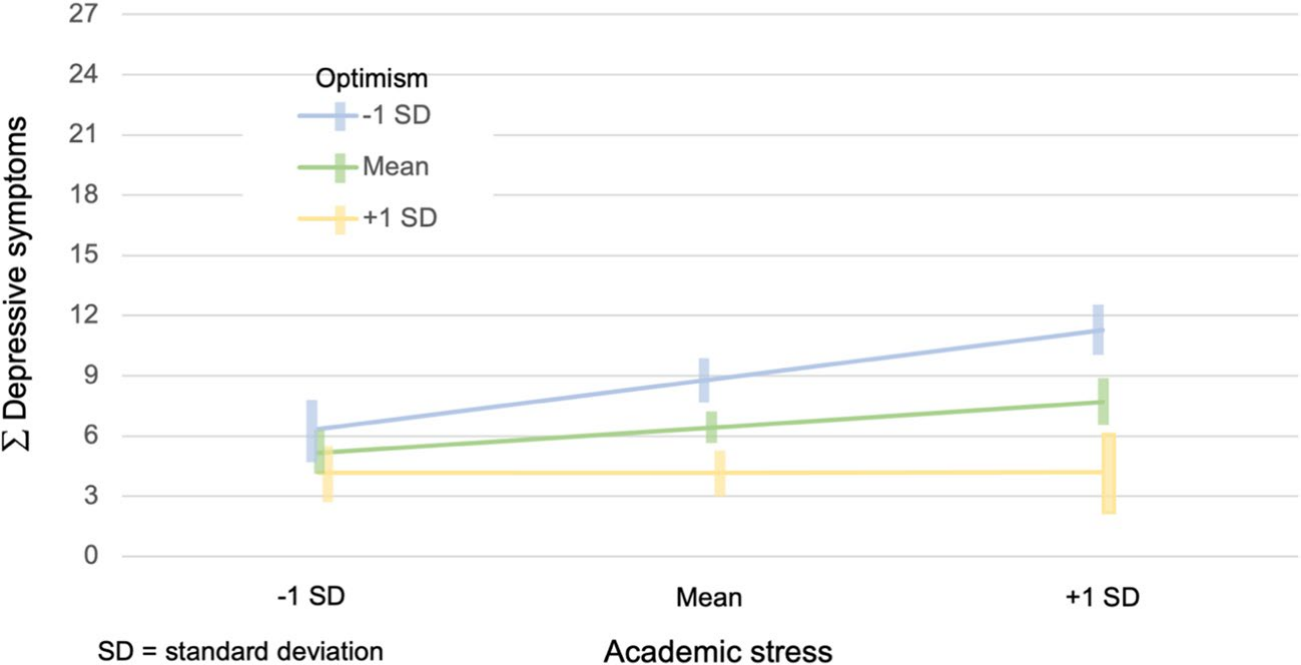
The moderating effect of optimism on the relationship between academic stress and depressive symptoms (FUM1, FUM2) in a mixed effects model with time as the random effect

The effects of optimism (hypothesis 2) and self-efficacy (hypothesis 3) on the relationship between academic stress and depressive symptoms are tested to evaluate them as individual moderating factors. In the second hypothesis, we expected optimism to function as a moderator. No significant results were found for hypothesis 2A (cross-sectional), as the regression analysis in PROCESS was neither significant for FUM1 (*p* = 0.140) nor for FUM2 (p = 0.109). Therefore, hypothesis 2A could not be confirmed. Using longitudinal data, Table 4 shows the fixed and random effects of the individual attributes (optimism and self-efficacy) on the association between academic stress and depressive symptoms. We found a significant interaction term for hypothesis 2B (longitudinal), which refers to the moderating role of optimism longitudinally (see Table 4 and Fig. 2). Hypothesis 2B could thus be confirmed.

**Fig. 3:**
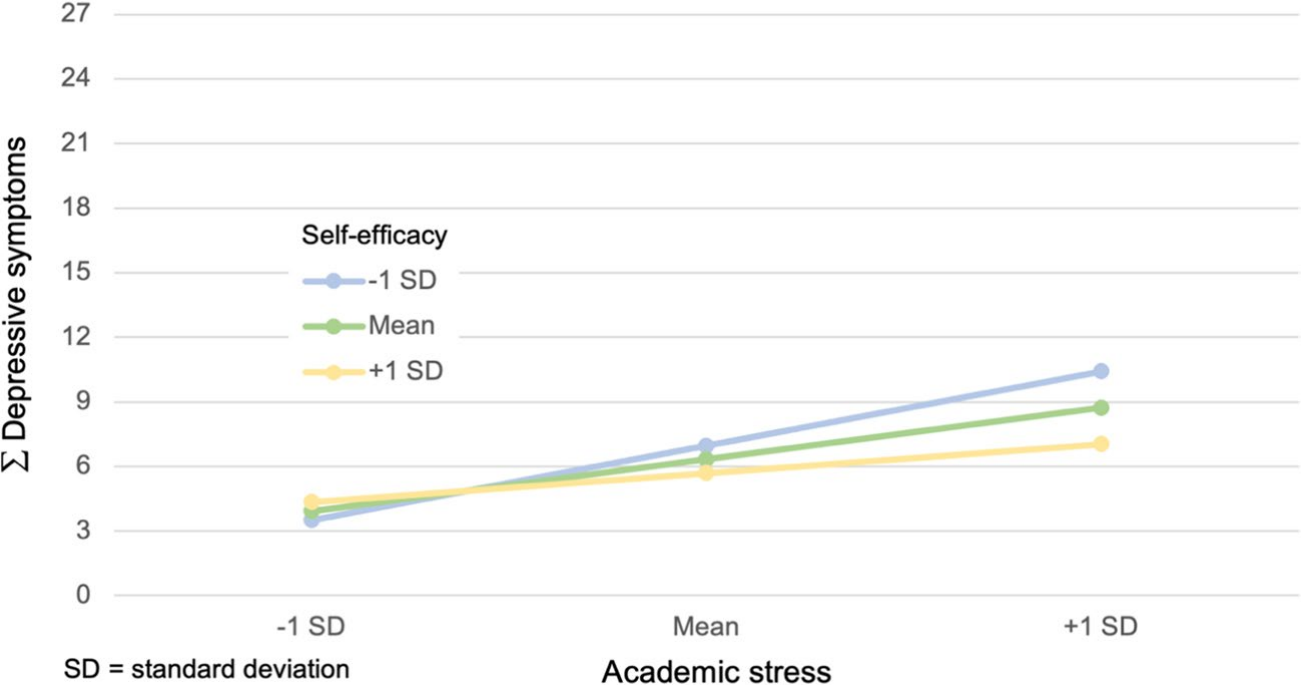
Effect of self-efficacy on the relationship between academic stress and depressive symptoms (FUM2)

In the third hypothesis, self-efficacy was analyzed as a moderator for the relationship between academic stress and depressive symptoms. Cross-sectionally, a significant result for self-efficacy as a moderator was found in FUM2 (F(3,103) = 15.2, p = 0.03, predicting 30.7% of the variance), but not in FUM1 (p = 0.74). That is why hypothesis 3A could only be confirmed for FUM2, as shown in Fig. 3.

The longitudinal analysis of self-efficacy as a moderator showed no significant results (see Table 4), hence hypothesis 3B could not be confirmed.

Research question 1 descriptively examines the knowledge about and use of the health promoting activities offered by the university. The results are shown in Table 5 for the different measurement points. Of the three categories in which the offers were divided, “university sports offers” was the most-used category for both FUM1 and FUM2. The other services, especially “psychological counseling”, were rarely used by the students, barring those with higher levels of academic stress (see Table 3). The correlation analysis in Table 3 also shows that the knowledge about study-related stress management offers was found to be associated with lower levels of depressive symptoms and more optimism.

**Table 5:** University health promotion offers

	Knowledge		Use	
	FUM1 n(%)	FUM2 n(%)	FUM1 n(%)	FUM2 n(%)
**University sports offers**				
Cross-sectional^1^	70 (66.0%)	77 (72.0%)	29 (27.4%)	26 (24.3%)
Longitudinal^2^	29 (67.4%)	30 (69.8%)	13 (30.2%)	12 (27.9%)
**Psychological counseling**				
**by the central university’s counseling service**				
Cross-sectional^1^	89 (84.0%)	83 (77.6%)	1 (0.9%)	5 (4.7%)
Longitudinal^2^	37 (86.0%)	38 (88.4%)	1 (2.3%)	3 (7.0%)
**by the university hospital’s counseling service**				
Cross-sectional^1^	25 (23.6%)	25 (23.4%)	-	1 (0.9%)
Longitudinal^2^	8 (18.6%)	8 (18.6%)	-	-
**Study-related stress management offers**				
**Course for stress and time management**				
Cross-sectional^1^	83 (78.3%)	82 (76.6%)	4 (3.8%)	4 (3.7%)
Longitudinal^2^	38 (88.4%)	36 (83.7%)	-	1 (2.3%)
**Mentoring**				
Cross-sectional^1^	79 (74.5%)	80 (74.8%)	3 (2.8%)	1 (0.9%)
Longitudinal^2^	32 (74.4%)	31 (72.1%)	2 (4.7%)	1 (2.3%)
**Psychoeducational information videos on learning**				
Cross-sectional^1^	61 (57.5%)	56 (52.3%)	18 (17.0%)	16 (15.0%)
Longitudinal^2^	20 (46.5%)	26 (60.5%)	10 (23.3%)	8 (18.6%)
**Self-assessment test on how to cope with study challenges**				
Cross-sectional^1^	7 (6.6%)	9 (8.4%)	-	-
Longitudinal^2^	1 (2.3%)	1 (2.3%)	-	-

*N* number of participants, *FUM1* follow-up measurement 1, *FUM2* follow-up measurement 2, ^1^ = FUM1, *n* = 106, *FUM2*, *n* = 107, ^2^*n* = 43

Research question 2 assesses the prevalence of academic stress and depressive symptoms during the first three semesters of medical training. Table 1 presents the data on depressive symptoms and academic stress at the three different measurement times BM (before the start of the pandemic), FUM1 and FUM2 (during the pandemic). As shown in Table 1, the two-week prevalence of any depressive syndrome in the last two weeks of BM was 4.9%, with an increase at FUM1 (25.5%) and FUM2 (23.4%). A slight increase of academic stress was observed in the first semester compared to the third semester (see Table 1).

## Discussion

The current study examined potential individual moderators and structural influencing factors in the context of the relationship between academic stress and depressive symptoms in medical students. After the investigation of the link between academic stress and depressive symptoms in the first semester (BM), we focused on optimism and self-efficacy as individual moderators, and the students’ knowledge and use of university health promotion offers as structural influencing factors in the following semesters (FUM1 and FUM2) to determine their possible influence on the development of stress and depressive symptoms. The survey was conducted three times during the first one and a half years of medical school, with the analyses being made both cross-sectionally and longitudinally. As had been expected, academic stress positively correlated with depressive symptoms cross-sectionally. The correlation analysis showed significant negative correlations between optimism and self-efficacy with academic stress. Furthermore, both individual factors were found to negatively correlate with depressive symptoms. Optimism moderated the association between academic stress and depressive symptoms in the longitudinal sample, while a similar effect of self-efficacy was found cross-sectionally for FUM2. The prevalence of depressive symptoms was found to increase during the study period (BM: 4.9%; FUM1: 25.5%; FUM2: 23.4%).

With respect to the first hypothesis, the more students reported being affected by academic stress, the more they also reported depressive symptoms for both the cross-sectional and longitudinal samples. The results here are similar to those of other studies that found a relationship between job stress and depressive symptoms [28, 65, 66]. Although the focus of those studies was workers and not medical students, the results appear to be transferable. One reason behind the association between academic stress and depressive symptoms may be the increased production of stress hormones [67], which alters brain activity, thereby causing cerebral disturbances. The demands of medical studies in general, with inordinately long study hours without time for recreation, recovery and balance, may be another possible explanation.

The population examined in our study included medical students in their first one and a half years of study. In this context, it must be borne in mind that the COVID-19 pandemic had started just a few months after the beginning of their studies.

It was not only the general population that was affected by the pandemic [68, 69], the well-being of students was also seriously impacted. Kaparounaki et al. (2020) [70] found students in Greece to be more anxious and depressed and experience more suicidal thoughts. Medical students from Switzerland experienced a greater feeling of social isolation during the pandemic along with decreased motivation, with the paucity of interactions with fellow students or professors negatively influencing their education [71]. There is some evidence that compulsory social isolation, as experienced during the COVID-19 pandemic, can increase depression symptomatology in medical students [72, 73], while social withdrawal can also appear as a symptom of depression. According to Achterbergh et al. (2020) [74], the relationship between depression and loneliness is a “vicious circle” as the two factors are mutually dependent and can reinforce each other. The changes toward online teaching, according to Zis et al. (2021) [75], had led to a decrease in mental health and increased cynicism. Bolatov et al. (2020) [76], on the other hand, found the prevalence of burnout syndrome, depression, anxiety and somatic symptoms to be lower in online learning programs compared to traditional learning among medical students in Kazakhstan. Thus, there are studies that also show certain advantages of online teaching. It is the start of medical school and the associated changes that have been found to have the greatest influence on mental health. Online teaching did not add additional stress to the examined first year medical students, according to Schindler et al. (2021) [77].

The survey date at the start of medical school can be an influencing factor with regard to the results. On the one hand, as is the case at the beginning of university or college education in general, the transition from life in the parental home is a crucial reason for high levels of stress. In addition, medical students have to contend with demanding academic challenges such as high workload or difficulties with time management from the beginning of their studies [45, 78].

As regards the second hypothesis, optimism moderates the association between academic stress and depressive symptoms longitudinally, but not cross-sectionally. In other studies, optimism was found to function as a moderator in the context of developing depression [28–30]. Finding a mild moderating effect of optimism on the relationship between stress and depression in employees, Banerjee (2012) [27] has posed a question as to whether optimism may be seen as a "separate general construct" from depression. It seems, therefore, that instead of only the inverse correlation between optimism and well-being, as is often described in the literature [24, 79], optimism also influences the association between academic related stress and depressive symptoms.

It is possible that optimism was not confirmed as a moderator cross-sectionally because the questionnaire used for all our analyses was a short version including only two items for optimism. More questions might have shed more light on the context. There is a longer version of the “SWOP” questionnaire with a total of 18 items instead of 9 (4 items to test optimism), although, as their inter-correlations indicate, they seem to be comparable in terms of content [80].

The results for hypothesis 3 show that self-efficacy functioned as a moderator in the cross-sectional sample in FUM2 (hypothesis 3A, shown in Fig. 3), but not in FUM1, and thus not longitudinally (hypothesis 3B). In other studies, self-efficacy has been seen as an important factor when it comes to preventing the development of psychological distress [33, 81–84]. That we found self-efficacy to be a moderator for the association between stress and depressive symptoms only in FUM2 might have been due to our relatively small study population. Since the correlations with the other variables (stress and depression) were as pronounced as expected, it can be assumed that a larger number of participants is needed for moderation. Future research should take this into consideration when testing self-efficacy as a moderator.

In comparison to other German medical students [2], the students in our study scored lower in optimism as well as in self-efficacy. It is plausible that their individual attributes were not as strong as those of their counterparts in the other study and therefore could not function as an inner resource. In addition, these individual factors might have been affected by the circumstances involving the pandemic, with distance learning, lockdowns and other daily restrictions likely reducing self-efficacy and the feeling of optimism. Despite the high levels of perceived stress and depression also reported by Heinen et al. (2017) [2] within their study sample, the enhancement of these individual attributes could be a point to be addressed by health promotion offers.

In general, the majority of students knew about all three groups of health promoting offers provided by the university. The knowledge about study-related stress management offers was found to be associated with lower levels of depressive symptoms and more optimism. Although stress management offers such as courses for stress and time management, or the mentoring program, were known by many students, the participation rate was still very low. Thus, there was a gap between the knowledge about health promoting offers and their use.

This is consistent with the gap between knowledge (or intention) and behavior [85] that describes the discrepancy between knowing what is good for one's (mental) health and behaving in a way that does not conform to that knowledge. This had already been investigated in contexts such as dietary changes [86] or smoking cessation [87]. The poor participation rates in the university health promotion offers may be partly due to time overlaps between these programs and mandatory courses in the students’ timetables (see supplementary Table 2, Additional File 2). Besides these overlaps, medical students typically have long to-do lists and rarely any free time. Givens and Tjia (2002) [46] and Tjia et al. (2005) [88] report that the most common obstacle to using counseling services is the lack of time. Thus, the participation in health promotion offers may be viewed by medical students as a “sacrifice” of their free time. It is important, therefore, that the benefit of such offers is made adequately clear.

The correlation analysis in Table 3 showed a positive relationship between knowledge about sports offers and academic stress for FUM1. This is an unexpected result, possibly a random correlation, as the one in FUM2 was not significant. It is possible, however, that students with higher stress levels tend to be more in search of opportunities that might help them cope with the stress they experience, which is probably why these students have more knowledge about the sports offers even though they do not use them.

As regards research question 2, the results show that the two-week prevalence of a depressive syndrome, which at BM was 4.9%, increased to 25.5% at FUM1 and to 23.4% at FUM2. This is relatively high compared to the results of other studies. Steffen et al. (2020) [89] found a prevalence of 15.7% for the general population in Germany, with a study by Hilger-Kolb et al. (2018) [90] showing a similar prevalence (15.8%) of depressive symptoms among German university students. Thus, it may be safe to assume that, compared to the general population of German university students, the medical students in the present sample have experienced depressive symptoms more frequently. Chow et al. (2018) [91] also found a 20.7% prevalence of depression among German medical students within one year of enrolment, which was higher than that of the general population. Interestingly, while at 12.9% the prevalence of depressive symptoms among medical students in Stockholm was found to be higher than the national average [92], it is significantly lower than our results. Miletic et al. (2015) [93] found a very high prevalence of depression among Serbian medical students, though it is in line with the general prevalence in Serbia. This shows that the results have to be seen in the context of the mental health situation of the respective country. In addition, the difficulties in the comparability of depressive symptoms may be due to the difference in measurement instruments and diversity among the student populations in the reported studies. It would also be interesting for future studies to investigate the aspect of the students' medical history in order to differentiate between those with a higher risk of developing depressive symptoms and those with a blank medical history.

Academic stress was found to arise when comparing the results of the first versus the third semester. In conformity with other studies using the MSSQ [15–17, 94], the academic related stressors (ARS) scored the highest, being the major source of stress for medical students in our study. The three items with the highest scores with regard to the perception of stress were “exams”, “high amount of study material to be learned” and “the lack of time for the revision of learned content”. These findings are consistent with those of Al-Qahtani et al. (2020) [15], who found the lack of time for revision and the large amount of study material followed by heavy workload and examinations to be the most relevant stressors among Saudi Arabian health profession students. Muhammad et al. (2019) [94] described the preclinical phase of study to cause more stress than the clinical phase. As our study observed only first-year medical students in their preclinical phase, this cannot be corroborated but would be interesting to investigate in future studies.

Our survey found the two-week prevalence of depressive symptoms to be at 4.9% at the beginning of the studies (BM), indicating that the students in our research had entered medical school with a much lower rate of depressive symptoms compared to what they experienced half a year (FUM1) and a year later (FUM2). This supports the observations of Brazeau et al. (2014) [95], Dyrbye and Shanafelt (2016) [96] and Hansell et al. (2019) [97], who found elevated levels of distress to arise only during medical training. The increase of psychological distress over the course of medical studies indicates that external stressors experienced within the study process likely affect students’ mental health. This must be countered with appropriate preventive measures such as health promotion activities offered by the university.

One practicable way to keep academic stress from leading to depressive symptoms would be to advocate optimism and self-efficacy through external health promotion offers. Other researchers have also considered the enhancement of optimism and self-efficacy as individual attributes as a useful means of health promotion [98–101]. As the medical students’ timetables are often tightly packed, the health promotion offers ought to be made available when it is possible for the students to take advantage of them. Online courses may prove useful in this regard as studies have shown that online activities were very well received during the COVID-19 pandemic [102] with a more efficient use of time [103].

This study has strengths as well as some weaknesses. One strength is the longitudinal study design. Tracking one semester at three different measurement points, it longitudinally analyzed the data of FUM1 and FUM2. The mixed effects model enabled us to examine the possible effect of time on our research and the stability of the effect over time. We did not only emphasize behavioral prevention, but also sought to identify possible ways to make university education more health promoting. Instead of making students contend with psychological distress on their own, attention needs to be paid to appropriately adjust the external circumstances by making useful and effective health promotion offers. Another interesting feature of this study is the special teaching concept of the study center, which includes more clinical practice and an earlier connection to clinical content compared to most other German medical schools.

The fact that medical students of only one university were included in the study, and that their study course followed a novel approach, thereby not being representative of general medical education in Germany, may be seen as a weakness of the study. To replicate our findings, future research would need to examine the knowledge and use of health promoting offers at different universities in Germany offering different medical study courses and health promotion programs. As regards our focus on the first year of study, while it may not be the most representative phase with respect to other stress factors such as new surroundings, and moving out of the parents’ house, these first semesters are important for the development of active coping strategies [93, 104, 105]. They are of interest especially with respect to possible preventive efforts and health promotion offers, which ought to start before the manifestation of depressive symptoms.

With regard to our cohort, the COVID-19 pandemic might have been another external influencing factor, adding additional stress. The switch to an online questionnaire from BM to FUM1 and FUM2 had resulted in a drop in the number of participants, especially for the longitudinal analysis. Thus, the relatively small sample size and the resulting low statistical power may be considered a weakness of our study. However, one advantage of a small sample size is that there is a lower probability of overestimating an effect [106]. Also, the few significant effects might be due to the relatively small sample size, but the effect sizes were medium to low, indicating that our analysis should be replicated [107, 108]. Another aspect that needs to be highlighted is that the pandemic had a major influence on the mental health of students worldwide [70, 75], which ought to be taken into consideration when interpreting our results, although the correlations between the variables (e.g., stress and depression) were pronounced as expected.

Furthermore, it is always difficult to measure stress, as stress questionnaires often cover strain in addition to psychological stress. The close relationship between the two makes it difficult to separate them.

It is possible that the students who participated in our study were particularly vulnerable to stress, resulting in a possible bias with respect to the types of students the study examined. However, as the comparison between the cross-sectional (0–24) and longitudinal study samples (1–18) in Table 2 shows, the students who were less depressed continued to take part in the study.

## Conclusion

This study gives first indications that individual factors such as optimism and self-efficacy have a moderating influence on the genesis of depressive symptoms in medical students. Global crises, such as a pandemic, can be an additional burden on students’ mental health. Highlighting the association between academic stress and depression, this study underscores the importance of health promotion offers, which can effectively mitigate the impact of academic stress on the mental health of medical students. As this study followed students only during their first three pre-clinical semesters, future research should investigate the students’ stress levels and the occurrence of depressive symptoms during the clinical phase of their study. Another research goal could be the examination of optimism and self-efficacy as moderators in the relationship between stress and depression in a larger study population.

## Supplementary Information

Below is the link to the electronic supplementary material.

Supplementary file1 (DOCX 22.4 KB)

Supplementary file2 (DOCX 19.7 KB)

Supplementary file3 (DOCX 17.3 KB)

## Data Availability

The datasets used and/or analyzed during the current study are available from the corresponding author on reasonable request.

## Abbreviations

BM: Baseline measurement
e.g.: Exempli gratia
FUM1: Follow-up measurement 1
FUM2: Follow-up measurement 2
M: Mean
Mdn: Median
n: Number of participants
PR: Prevalence rate
Ref: Reference
SD: Standard Deviation
