# Supplementary material for: The Role of Optimism and Self-Efficacy in the Relationship between Academic Stress and Depressive Symptoms in Medical Students Including the Use and Knowledge of Structural Health Promotion Offers
Source: Med Sci Educ. 2024 Dec 18;35(2):807–22. doi: 10.1007/s40670-024-02240-4 (PMC12058625; doi:10.1007/s40670-024-02240-4)
Supplement: Supplementary file 1 — Supplementary file1 (DOCX 22.4 KB) [file 40670_2024_2240_MOESM1_ESM.docx]

**Additional file 1**

**The role of optimism and self-efficacy in the relationship between academic stress and depressive symptoms in medical students including the use and knowledge of structural health promotion offers**

*Authors:*

Annika Arnold^1^, Petra Maria Gaum^1^, Jessica Lang^1^

^1^Institute for Occupational, Social and Environmental Medicine, Medical Faculty, RWTH Aachen University, Aachen, Germany

*Corresponding author:*

Annika Arnold

E-Mail: annika.arnold@rwth-aachen.de

*Journal:*

Medical Science Educator

**Correlation analysis (Spearman-Rho) of demographic variables for the three measurement points**

**Supplementary Table 1.1**: Baseline measurement

|  | **1)** | **2)** | **3)** | **4)** |
| --- | --- | --- | --- | --- |
| **1) Sex** | 1.000 | -0.126 | 0.063 | .222^**^ |
| **2) Age** | -0.126 | 1.000 | -.197^**^ | -0.101 |
| **3) PHQ (sum scale)** | 0.063 | -.197^**^ | 1.000 | .249^**^ |
| **4) Academic stress** | .222^**^ | -0.101 | .249^**^ | 1.000 |

n= number of participants; p-value (significance; two-tailed): + = .05 < p-value <.1; * p-value <.05; ** p-value <.01; n= 226

**Supplementary Table 1.2**: Follow-up measurement 1

|  | **1)** | **2)** | **3)** | **4)** | **5)** | **6)** |
| --- | --- | --- | --- | --- | --- | --- |
| **1) Sex** | 1.000 | -0.012 | 0.173 | .210^*^ | -0.136 | -.423^**^ |
| **2) Age** | -0.012 | 1.000 | 0.100 | -0.017 | -0.008 | -0.047 |
| **3) PHQ (sum scale)** | 0.173 | 0.100 | 1.000 | .630^**^ | -.456^**^ | -.361^**^ |
| **4) Academic stress** | .210^*^ | -0.017 | .630^**^ | 1.000 | -.384^**^ | -.541^**^ |
| **5) Optimism** | -0.136 | -0.008 | -.456^**^ | -.384^**^ | 1.000 | .481^**^ |
| **6) Self-efficacy** | -.423^**^ | -0.047 | -.361^**^ | -.541^**^ | .481^**^ | 1.000 |

n= number of participants; p-value (significance; two-tailed): + = .05 < p-value <.1; * p-value <.05; ** p-value <.01; n= 106

**Supplementary Table 1.3**: Follow-up measurement 2

|  | **1)** | **2)** | **3)** | **4)** | **5)** | **6)** |
| --- | --- | --- | --- | --- | --- | --- |
| **1) Sex** | 1.000 | 0.083 | 0.097 | .227^*^ | -0.189 | -0.166 |
| **2) Age** | 0.083 | 1.000 | 0.007 | 0.072 | -0.027 | -0.056 |
| **3) PHQ (sum scale)** | 0.097 | 0.007 | 1.000 | .496^**^ | -.399^**^ | -.374^**^ |
| **4) Academic stress** | ,227^*^ | 0.072 | .496^**^ | 1.000 | -.367^**^ | -.514^**^ |
| **5) Optimism** | -0,189 | -0.027 | -.399^**^ | -.367^**^ | 1.000 | .431^**^ |
| **6) Self-efficacy** | -0,166 | -0.056 | -.374^**^ | -.514^**^ | .431^**^ | 1.000 |

n= number of participants; p-value (significance; two-tailed): + = .05 < p-value <.1; * p-value <.05; ** p-value <.01; n= 107
