# Supplementary material for: The Role of Optimism and Self-Efficacy in the Relationship between Academic Stress and Depressive Symptoms in Medical Students Including the Use and Knowledge of Structural Health Promotion Offers
Source: Med Sci Educ. 2024 Dec 18;35(2):807–22. doi: 10.1007/s40670-024-02240-4 (PMC12058625; doi:10.1007/s40670-024-02240-4)
Supplement: Supplementary file 2 — Supplementary file2 (DOCX 19.7 KB) [file 40670_2024_2240_MOESM2_ESM.docx]

**Additional File 2**

**The role of optimism and self-efficacy in the relationship between academic stress and depressive symptoms in medical students including the use and knowledge of structural health promotion offers**

*Authors:*

Annika Arnold^1^, Petra Maria Gaum^1^, Jessica Lang^1^

^1^Institute for Occupational, Social and Environmental Medicine, Medical Faculty, RWTH Aachen University, Aachen, Germany

*Corresponding author:*

Annika Arnold

E-Mail: annika.arnold@rwth-aachen.de

*Journal:*

Medical Science Educator

**Supplementary Table 2:
Overlaps between the health promotion offers and the students' timetable**

|  | Would there be any overlaps with the timetable if you wanted to take advantage of the offers? | | How often have you wanted to use the offers and could not because of overlaps with the timetable? | |
| --- | --- | --- | --- | --- |
|  | **FUM1** | **FUM2** | **FUM1** | **FUM2** |
| No, never | 25 (23.6%) | 20 (18.7%) | 52 (49.1%) | 62 (57.9%) |
| Yes, rarely (1-2x) | 30 (28.3%) | 14 (13.1%) | 40 (47.7%) | 29 (27.1%) |
| Yes, often (3-6x) | 14 (13.4%) | 16 (15.0%) | 13 (12.3%) | 11(10.3%) |
| Yes, very often (>6x) | 4 (3.8%) | 10 (9.3%) | 1 (0.9%) | 5 (4.7%) |
| I don’t know | 33 (31.1%) | 47 (43.9%) | - | - |
|  |  |  |  |  |

n= number of participants; FUM1= follow-up measurement 1; FUM2= follow-up measurement 2; n_FUM1_=106, n_FUM2_=107
