# Supplementary material for: The Role of Optimism and Self-Efficacy in the Relationship between Academic Stress and Depressive Symptoms in Medical Students Including the Use and Knowledge of Structural Health Promotion Offers
Source: Med Sci Educ. 2024 Dec 18;35(2):807–22. doi: 10.1007/s40670-024-02240-4 (PMC12058625; doi:10.1007/s40670-024-02240-4)
Supplement: Supplementary file 3 — Supplementary file3 (DOCX 17.3 KB) [file 40670_2024_2240_MOESM3_ESM.docx]

**Additional File 3**

**The role of optimism and self-efficacy in the relationship between academic stress and depressive symptoms in medical students including the use and knowledge of structural health promotion offers**

*Authors:*

Annika Arnold^1^, Petra Maria Gaum^1^, Jessica Lang^1^

^1^Institute for Occupational, Social and Environmental Medicine, Medical Faculty, RWTH Aachen University, Aachen, Germany

*Corresponding author:*

Annika Arnold

E-Mail: annika.arnold@rwth-aachen.de

*Journal:*

Medical Science Educator

**Supplementary Table 3: Drop out analysis of study participants overt the three measurement points - Chi Square Statistic**

|  | **Participants at BM: Dropouts** | **Participants at BM and FUM1 or FUM2** | **F/Chi Square** | **p** |
| --- | --- | --- | --- | --- |
| **Total n in BM: 242** | 129 | 113 |  |  |
| **Age** | 21.21 (3.97) | 20.55 (3.22) | 1.98 | 0.16 |
| **Gender** | Male 48 (37.2%)  Female 81 (62.8%) | Male 33 (29.5%)  Female 79 (70.5%) | 1.61 | 0.21 |
| **Academic stress** | 1.21 (0.56) | 1.24 (0.55) | 0.11 | 0.74 |
| **PHQ (sum scale)** | 3.45 (3.12) | 3.55 (3.56) | 0.05 | 0.08 |
| **Major depressive syndrom** | 1 (0.8%) | 3 (2.7%) | 1.26 | 0.26 |
| **Other depressive syndrom** | 2 (1.6%) | 3 (2.7%) | 1.70 | 0.93 |
|  |  |  |  |  |

n= number of participants; BM= Baseline measurement; FUM1= follow-up measurement 1; FUM2= follow-up measurement 2
